# Level of traffic stress-based classification: A clustering approach for Bogotá, Colombia

**DOI:** 10.1016/j.trd.2020.102420

**Published:** 2020-08

**Authors:** Jorge A. Huertas, Alejandro Palacio, Marcelo Botero, Germán A. Carvajal, Thomas van Laake, Diana Higuera-Mendieta, Sergio A. Cabrales, Luis A. Guzman, Olga L. Sarmiento, Andrés L. Medaglia

**Affiliations:** a Centro para la Optimización y Probabilidad Aplicada (COPA), Departamento de Ingeniería Industrial, Universidad de los Andes, Bogotá, Colombia; b Fundación Despacio, Colombia; c Facultad de Medicina, Universidad de los Andes, Bogotá, Colombia; d Grupo de Estudios en Sostenibilidad Urbana y Regional (SUR), Departamento de Ingeniería Civil y Ambiental, Universidad de los Andes, Bogotá, Colombia

**Keywords:** Cycling, Level of Traffic Stress, Cluster Analysis, Latin America, Bogotá

## Abstract

The Level of Traffic Stress (LTS) is an indicator that quantifies the stress experienced by a cyclist on the segments of a road network. We propose an LTS-based classification with two components: a clustering component and an interpretative component. Our methodology is comprised of four steps: (i) compilation of a set of variables for road segments, (ii) generation of clusters of segments within a subset of the road network, (iii) classification of all segments of the road network into these clusters using a predictive model, and (iv) assignment of an LTS category to each cluster. At the core of the methodology, we couple a classifier (unsupervised clustering algorithm) with a predictive model (multinomial logistic regression) to make our approach scalable to massive data sets. Our methodology is a useful tool for policy-making, as it identifies suitable areas for interventions; and can estimate their impact on the LTS classification, according to probable changes to the input variables (e.g., traffic density). We applied our methodology on the road network of Bogotá, Colombia, a city with a history of implementing innovative policies to promote biking. To classify road segments, we combined government data with open-access repositories using geographic information systems (GIS). Comparing our LTS classification with city reports, we found that the number of bicyclists’ fatal and non-fatal collisions per kilometer is positively correlated with higher LTS. Finally, to support policy making, we developed a web-enabled dashboard to visualize and analyze the LTS classification and its underlying variables.

## Highlights

• Data-informed methodology calculates the level of traffic stress of cyclists.
• Method scales to massive data sets by coupling a classifier with a predictive model.
• Methodology tested on the road network of Bogotá (Colombia)
• Web-enabled dashboard supports policy making and interventions to reduce stress.
• Number of bicyclists’ collisions per kilometer correlates with higher stress.

## 1 Introduction

The Level of Traffic Stress (LTS) is an indicator that classifies the components of a road network according to the stress experienced by cyclists (P. G. Furth et al., 2016, Mekuria et al., 2012). The original LTS indicator classifies every road segment in a range from 1 to 4 using decision trees (Murphy and Owen, 2019), based on 21 variables related to bicycle infrastructure, roadway size and layout, and intersection characteristics. The original four categories of LTS correspond to the road conditions tolerable to the four types of cyclists proposed by Geller (2006). Segments classified as LTS 1 are assumed to be comfortable for all types of cyclists while LTS 4 segments are suitable only for the most experienced cyclists; segments at intermediate levels of LTS are considered appropriate for cyclists with moderate experience. As the former group forms the majority of the population (Dill and McNeil, 2013, Dill and McNeil, 2016), infrastructure interventions aimed at reducing stress and creating low-stress networks are crucial in creating the conditions under which modal share can increase.

Consequently, the LTS indicator has been used in various cities to identify and prioritize infrastructure interventions with the goal of creating an interconnected network of low-stress routes (Bearn, 2015, Berkeley City Council, 2017. G. Furth et al., 2016, Mekuria et al., 2012, Moran et al., 2018, Murphy and Owen, 2019, Pérez et al., 2017, Prabhakar and Rixey, 2017, Semler et al., 2017, Vogt and Rachel, 2015). The indicator can contribute to planning processes by: (1) approximating cycling safety conditions at a city level; (2) identifying missing links in the low-stress network; (3) evaluating the benefits of new infrastructure; and (4) prioritizing infrastructure investment to maximize impact and avoid wasteful spending.

Due to the economic, social, and cultural context in Latin America, the urban plans have produced a limited cycling infrastructure in some of the cities of the region (Guerra, Caudillo, Monkkonen, & Montejano, 2018). Nevertheless, many Latin American cities have implemented innovative, equitable, and sustainable transportation policies that promote safe bicycling (Ríos et al., 2015, Rodríguez et al., 2017a, Rodríguez et al., 2017b, Rodríguez et al., 2017a, Rodríguez et al., 2017b). However, very few planning agencies have applied LTS or other stress prediction methodologies to improve planning practice; and academic studies are similarly scarce (Tucker & Manaugh, 2018). A major impediment to the adoption of LTS is that, although most of the criteria commonly used for classification (such as traffic volume and roadway width) may apply universally, the parameters and thresholds used in different contexts may not account for specific characteristics of Latin American cities and their road networks. Considering the great challenges in terms of cyclist safety faced by the cities in the region (Carvajal et al., 2020, Hidalgo and Huizenga, 2013), the potential of LTS-based data-informed tools to improve planning is not to be understated.

One of the greatest advantages of the LTS is its simplicity, based on a transparent decision tree approach in most of its implementations. However, LTS can be challenging to implement and interpret due to numerous segment-level variables necessary for the classification (Harvey, Kevin Fang, & Rodriguez, 2019). These challenges have motivated the development of alternative LTS classification methods that use fewer and more commonly-available variables (Conveyal, 2015; P. Furth, 2017, Lowry et al., 2016, Montgomery County Planning Department, 2018, Oregon Department of Transportation, 2017, People For Bikes, 2017). As a result, some of these modifications to the LTS methodology produce different LTS categories (i.e., LTS High, LTS Low) (Montgomery County Planning Department, 2018, People For Bikes, 2017), but more importantly, all these alternative LTS classification methods hardly produce the same results (Harvey et al., 2019). Thus, ways to classify the road network that reflect the potential stress experienced by cyclists is still an open discussion topic.

This paper presents a data-informed LTS-based road classification methodology that relies upon a clustering component combined with statistically calibrated models and readily available road network data. The clustering component presents a fast and efficient way to differentiate similar segments of the road network. The interpretative component accounts for LTS label assignment (to the road segments) taking into account the place-specific context. Our approach is city-wide scalable without requiring fieldwork to collect input data for the classification. We apply our LTS-based classification methodology in the road network of Bogotá (Colombia), introducing LTS categories *Low, Medium*, *High*, and *Extremely high,* consistent with the characteristics of this Latin American metropolis. Finally, we compare our LTS results with the cyclists’ collisions reported in Bogotá during 2017; and find that our data-informed LTS-based road classification results are consistent with the literature that relates LTS to collisions.

This paper is organized as follows. Section 2 presents a literature review of different methodologies to calculate a wide variety of cycling-related metrics including LTS, as well as the common variables used in these methodologies. Section 3 presents our data-informed LTS-based classification methodology. Section 4 presents the case study of Bogotá, which illustrates the applicability of our methodology. Finally, Section 5 concludes the paper and outlines ongoing and future work.

## 2 Literature review

Planning strategies for improvements to cycling conditions can be divided into demand- and supply-based methodologies. A demand-based planning approach bases decisions on existing (and in some cases, potential) travel behavior, particularly through the study and quantification of trip flows from one area to another (Landis, 1996, Rybarczyk and Wu, 2010, Schwartz et al., 1999, Turner et al., 1997). Such methodologies are useful to identify improvements to benefit the most cyclists by focusing on demand rather than road conditions. Nevertheless, these models cannot identify what improvements are necessary at specific sites (Hyodo et al., 2000, Porter et al., 1999, Schwartz et al., 1999). Moreover, demand is not fixed, and likely depends on the road conditions faced by cyclists. For instance, several studies have argued that cyclists base their route choice decisions on the *level of stress* experienced on the road network (Furth et al., 2016, Geller, 2011, Landis, 1996). A supply-based planning approach focuses on analyzing the conditions of the road network for cyclists, and generally prioritizes improvements where conditions are worst, leading to implementation of protected infrastructure along all major arterials and collectors (Harkey et al., 1998, Landis, 1996, Rybarczyk and Wu, 2010). Among supply-based models, the LTS indicator has been popularized, standing out for being based on a simple calculation with readily available data. Although supply-based planning methodologies differ in terms of their indicators’ calculation approach, all of them identify a set of variables that can affect cyclists. Depending on the variables taken into account, supply-based methodologies can be classified into three main categories, focusing on: (i) road and traffic conditions, (ii) perceived factors, and (iii) physiological factors.

Methodologies based on road and traffic conditions classify each road segment (and in some cases, intersections) into different groups with similar characteristics based on selected variables. The Davis Bicycle Safety Index (Davis, 1987) was one of the first systematic attempts to measure the operational conditions of roads for cyclists. It calculates the Roadway Segment Index (RSI) and the Intersection Evaluation Index (IEI) to produce the Bicycle Safety Index Rating (BSIR), based on relatively easy to measure road characteristics. Sorton and Walsh (1994) were the first to focus on “stress” and created the Bicycle Stress Level (BSL) that categorizes each road segment into one of five levels of stress. Landis (1996) calculated the Interaction Hazard Score (IHS), which identifies six categories for the level of service using existing road and traffic variables. LTS, introduced by Mekuria et al. (2012), identifies four categories of traffic stress based on threshold criteria of road characteristics, with the lowest performing criterion determining classification. Lowry et al. (2016) calculated bicycle stress based on Marginal Rates of Substitution (MRS) for every road segment based on stress-creating and stress-reducing factors. In sum, the majority of the methodologies based on road and traffic conditions use precise and readily available data. However, many of these methodologies use subjective non-statistically calibrated decision criteria (B. Landis, Vattikuti, & Brannick, 1997).

In contrast, methodologies based on perceived factors use surveys and real-time perception of cyclists to identify different metrics for the road, such as: the stress generated, its level of service, or even its latent demand (in demand-based models). These perceptions are then matched to a specific set of variables present in each road. Sorton and Walsh (1994) validated their BSL with a group of cyclists who rated segments based on the traffic conditions captured on videos. Landis et al. (1997) introduced the Bicycle Level of Service (BLOS), to consider a statistically calibrated model to describe the level of service of a road. It uses a mathematical function of human perception of stimuli, based on observations of a large group of cyclists, thus identifying stress factors and translating them to an index that is divided into six stress categories. Using a similar approach, Harkey et al. (1998) constructed the Bicycle Compatibility Index (BCI). They showed videos of different segments to a group of cyclists and asked them how comfortable they would feel under those operational conditions. Winters et al. (2013) introduced the Bikeability Index, a mathematical equation that describes bikeability based on a survey, travel behavior studies, and focus groups. Finally, Blanc and Figliozzi (2016) describe cyclists’ comfort levels as a function of bicycle infrastructure with an ordinal logistic regression model. Methodologies in this branch relate qualitative data on perceived factors with road characteristics using statistical models. However, input data often require large surveys with diverse focus groups.

Finally, methodologies based on physiological factors use technology to measure biological responses to conditions experienced by cyclists. Caviedes and Figliozzi (2018) base their methodology on on-road measurements of physiological stress by means of the Galvanic Skin Response (GSR) of cyclists exposed to different traffic conditions. This methodology allows for a physiological measurement of stress on a previously determined route. A similar approach is used by Berger and Dörrzapf (2018), who apply bio-physiological sensors and empirical data to measure stress on a previously determined route. However, physiological approaches can only identify conditions that affect cycling stress along the specific route measured, and it does not necessarily follow that those relationships hold in other environments, particularly where road conditions or social norms may be substantially different. In addition, the use of these approaches to calculate a bikeability index for an entire urban area to support planning policies may be time, cost, and human resource intensive, making such an index subject to the availability of funds and institutional capacity.

Without exception, the methodologies presented above were developed in places with specific characteristics that shaped the decision criteria in the stress or bikeability classification (Geller, 2006, Mekuria et al., 2012, Winters et al., 2013). Some adaptations of these methodologies have been carried out for other cities with similar contexts (Caviedes and Figliozzi, 2018, Duthie et al., 2014, Krenn et al., 2015, Mueller and Hunter-Zaworski, 2014, Murphy and Owen, 2019, Rybarczyk and Wu, 2010). Most of the LTS implementations that have appeared in the literature have been adapted to match place-specific characteristics from where they have been conceived. Lowry et al. (2016) adapted the original LTS to match specific characteristics of Seattle, WA (US), due to the local agency-collected data they used. Montgomery County Planning Department (2018) modified the original LTS classification outcome and introduced LTS 0, LTS 2.5, and LTS 5 categories to match the specific needs of the Montgomery County in Maryland (US). Modifications to the original LTS were also proposed by Oregon Department of Transportation (2017) in Oregon (US) and by Berkeley City Council (2017) in Berkeley, CA (US). Vogt (2015) adapted LTS to compare its relationship with bicycle collisions in four cities in New Hampshire (US). Pérez et al., 2017, Semler et al., 2017 used different LTS adaptations in Washington D.C. (US). Some other examples of LTS modifications are common (Bearn, 2015, Moran et al., 2018, Prabhakar and Rixey, 2017). These place-specific modifications to original LTS classification in the previous implementations are guided to match the characteristics of the cities where they have been applied, therefore, these modifications may not be applicable to other cities with a different economic, social, and cultural context.

On the other hand, other LTS implementations have been produced to overcome the place-specificity and generate a more general LTS. Furth (2017) aimed to improve the LTS generalizability by producing its “2.0” version, using only six of the original 21 variables. In an effort to provide high-level analyses, Conveyal (2015) partnered with The World Bank Group and presented an extreme simplification of traditional LTS, which they called “Surrogate LTS”, using only four variables derived from a few tags in OpenStreetMap (OSM) (OpenStreetMap contributors, 2018). Data for these tags is not complete in many cities, and the parameters and thresholds defined may not account for the characteristics of other cities. People For Bikes (2017) modified the original LTS classification using few variables and produced a two-level scale (low and high stress) that reflect the imprecision of their inputs. Tucker and Manaugh (2018) use an extreme simplification of LTS using only one variable retrieved from OSM labels. These general implementations of the LTS use very few variables that hardly capture the stress factors in multiple road networks in different contexts. Moreover, all of these implementations of LTS, both general and place-specific, hardly produce the same LTS classification. Harvey et al. (2019) used seven of the most-well known LTS implementations (Conveyal, 2015; P. Furth, 2017, Lowry et al., 2016, Mekuria et al., 2012, Montgomery County Planning Department, 2018, Oregon Department of Transportation, 2017, People For Bikes, 2017) to classify the road networks of Portland, OR (US) and Austin, TX (US) obtaining a wide variety of LTS results. Therefore, it becomes unclear which of these adaptations of LTS reflect, if any of them can do so, the characteristics of cities with different economic, social, and cultural contexts.

Counter to the initial conception of LTS, which emphasized easily obtainable variables related to physical and traffic conditions, some variables used in the methodologies presented above may not be easy to obtain or calculate. Examples of such variables are trip purpose, commercial driveways per mile, location factors, road connectivity, bicycle-friendly roads, slope, mode sharing, bike commuting, uncontrolled vehicular access, cross traffic generation, vehicles turning into driveways, vehicles pulling in or out of parking, pavement factors, green and aquatic areas, route comfort, or route frequency (Blanc and Figliozzi, 2016, Harkey et al., 1998, Krenn et al., 2015, Landis et al., 1997, Landis, 1996, Mingus, 2015, Winters et al., 2013). On the other hand, some of the variables used in many of the methodologies presented above are of utmost importance in some contexts but may not be relevant in other cities due to cultural differences. For example, right-turn lanes are not common in most Latin American cities, while they are widespread in North America. Nevertheless, a small set of relevant variables commonly used in the methodologies previously reviewed are readily available in many countries. Among these variables are the road width, number of lanes, presence of cycling infrastructure, presence of heavy vehicles, traffic speed, and traffic volume (Davis, 1987, Epperson, 1994, Harkey et al., 1998, Krenn et al., 2015, Landis et al., 1997, Landis, 1996, Lowry et al., 2016, Mingus, 2015, Sorton and Walsh, 1994).

In summary, methodologies based on road and traffic conditions use precise and readily available data, but rely on subjective or non-replicable decision criteria to produce measures of stress. Methodologies based on perceived stress relate real cyclists’ observations to road characteristics through robust statistical models, however, they require data that is usually difficult or costly to obtain. Finally, methodologies based on physiological stress effectively relate cycling conditions to actually-experienced stress but are expensive and may not be easy to extrapolate to determine stress conditions at a metropolitan level. All of these methodologies have been designed in urban contexts that reflect place-specific characteristics that are not necessarily the same for other cities. Finally, the proliferation of LTS adaptations that overcome both place-specificity or generality requirements produce different LTS classifications over the same road networks. In this paper, we respond to these gaps by proposing a data-informed LTS-based classification methodology that relies on a clustering component that uses statistically calibrated models with precise and readily available roadway data to differentiate segments in a road network; and an interpretative component that classifies similar segments under the LTS prism considering the place-specific context and using universal LTS-related criteria.

## 3 Methodology

**Fig. 1:**
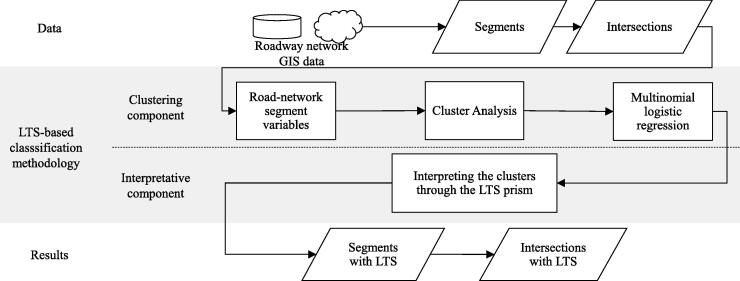
General overview of the data-informed LTS-based classification methodology.

Fig. 1 presents the flow of our data-informed LTS-based classification methodology for a given road network. At the top, the data layer relies heavily on GIS transformations and processing to build the set of segments and intersection variables of the road network required for the clustering component of the methodology. Following the calculation of the variables, we feed a cluster analysis with a representative subset of the network to classify segments with similar characteristics. In Section 4.3 we present a statistical analysis of the results of our LTS-based classification methodology when using different representative subsets. With the results of the classifier, we train a multinomial logistic regression that predicts the likelihood of a (new) segment to belong to a given cluster. Having classified all the segments into different clusters, the interpretative component of the methodology assigns an LTS category to each cluster based on the available relevant statistics and the place-specific context. Finally, we classify intersections based on the LTS values of intersecting segments.

### 3.1 Road-network segment variables

We use eight variables considered as stress factors for cyclists to classify the road network segments into different clusters. These variables are present in most of the LTS methodologies reviewed in Section 2. Aside from being readily available and easy to calculate, the set of variables is comprehensive: three capture the physical factors of the road segments, one captures the traffic mix, and four capture the traffic conditions. Table 1 presents the eight variables.

**Table 1:** Variables used in the LTS-based methodology.

Variable	Factor	Type
Roadway width	Physical	Continuous
Number of lanes	Physical	Discrete
Presence of cycling infrastructure	Physical	Categorical
Presence of heavy vehicles	Traffic mix	Categorical
Vehicles' speed	Traffic	Continuous
Traffic density	Traffic	Continuous
Traffic flow	Traffic	Continuous
Congestion index	Traffic	Continuous

Here, we present a brief description of the eight variables, starting with the four variables that describe the road segments and their built environment:

• ***Roadway width** (*w*)*: Continuous variable that represents the width of a road segment, measured as the distance from one edge of the road to the other.
• ***Number of lanes** (*l*)*: Discrete variable that represents the number of single-vehicle lines of traffic in a given segment.
• ***Presence of cycling infrastructure** (*b*)*: This variable indicates the presence of cycling infrastructure along the road segment. It could be a categorical variable indicating the type of infrastructure present or, given data limitations, it could be a binary variable indicating the presence or absence of infrastructure.
• ***Presence of heavy vehicles** (*h*)*: Binary variable that indicates whether the local administration allows transit of heavy vehicles such as trucks or buses.

In the transportation planning literature, traffic is commonly explained by the relationship among four variables: speed, traffic density, traffic flow, and congestion (Wang, Quddus, & Ison, 2013). To calculate all these variables for a specific road segment we used well-known formulas in the transportation planning field. These formulas rely on the average and free-flow traversing times t and $t_{0}$, respectively. In Section 4.1 we detail the data process conducted to obtain these times programmatically. The traffic variables follow:

• ***Vehicles’ speed** (*v*)*: Continuous variable that denotes the average speed of motorized vehicles traversing a road segment. We obtained this variable by dividing the length of the road by its average traversing time.
• ***Traffic density** (*k*)*: Continuous variable that indicates the average number of vehicles in the road segment per unit length. Classic traffic density formulas in the traffic planning literature have been proposed by Greenshields et al., 1935, Greenberg, 1959, Underwood, 1961, and Drake et al. (1967), among others. To calculate traffic density we use the Gaussian method (Drake et al., 1967):
  (1) $$k=k_{0}{\left(2\ln\left(\frac{v_{0}}{v}\right)\right)}^{\frac{1}{2}},$$
  where $k_{0}$ is the standstill traffic density, that is, the number of standstill vehicles in a roadway per unit length; and $v_{0}$ is the free-flow speed obtained with the free-flow traversing time $t_{0}$.
• ***Traffic flow** (*q*)*: Continuous variable that captures the number of vehicles traversing a road segment per time unit. To calculate this variable we use the universal traffic flow formula (Treiber and Kesting, 2013, chap. 7):
  (2) q=kv
  where k is traffic density and v the vehicles’ speed, described above.
• ***Congestion** (*c*)*: Continuous variable that describes the level of traffic congestion in a road segment. To calculate this variable, we use the congestion index, firstly proposed by Richardson and Taylor (1978). This index represents the average delay a regular vehicle experiences along the segment, compared against the time it takes to traverse the segment at free-flow. To calculate it we used the following formula:
  (3) $$c=\frac{t-t_{0}}{t_{0}}$$

We use these eight variables as the input of a cluster analysis that identifies the groups of road segments with similar characteristics. Nevertheless, other factors may be included, depending on data availability or context.

### 3.2 Cluster analysis

**Fig. 2:**
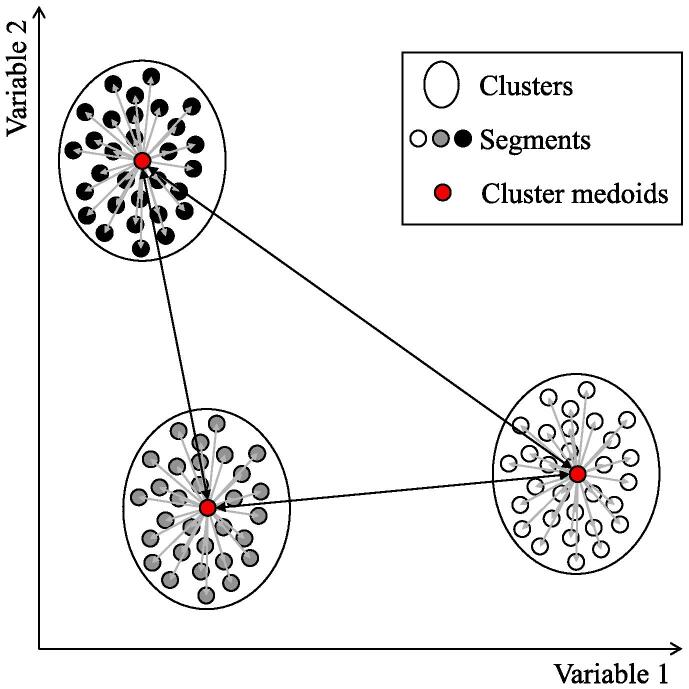
Cluster analysis intuition.

With cluster analysis we seek to find groupings of road segments based on the variables described in Section 3.1. Segments within a cluster should be both *similar* internally and different to those in different clusters. To illustrate this step of our methodology, let us consider the example shown in Fig. 2, which plots road segments with two characteristics (i.e., variables). In this illustrative example, we can visually identify three clusters based on these two variables. Segments within a given cluster share similar traits and are different from those in the other two clusters.

**Fig. 3:**
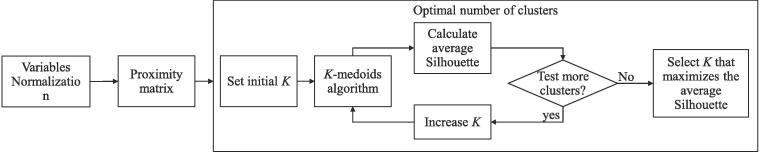
Cluster analysis overview.

Fig. 3 presents an overview of our cluster analysis that finds groups of road segments in higher dimensions (i.e., multiple variables). First, we normalize the variables. Then, we calculate a proximity matrix between every pair of road segments. At the core of our cluster analysis, we use a *K*-medoids algorithm (Kaufman & Rousseeuw, 1990) that groups similar segments into *K* clusters which are represented by their corresponding *medoid* (Ester, Kriegel, Sander, & Xu, 1996).

I. ***Variables’ normalization***: we normalize the variables of the road segments to avoid bias in the comparison process due to scale differences (Larose, 2005). In particular, we use the z-scoring method to transform the continuous variables while keeping their original distributional form. We use this method to normalize the segments’ width (w), number of lanes (l), vehicles speed (v), traffic density (k), traffic flow (q), and congestion index (c).
II. ***Proximity matrix***: the input for the *K*-medoids algorithm is a proximity matrix that determines the similarity between each pair of road segments (Hastie, Tibshirani, Friedman, & Franklin, 2005). We calculate this degree of similarity using Gower’s distance, which is suitable under the presence of continuous and discrete variables (Gower, 1971).
III. ***K-medoids algorithm***: this algorithm assigns all elements (i.e., road segments) to *K* clusters in such a way that it minimizes the distance between the medoid and each element within the cluster (see grey lines within clusters in Fig. 2), while simultaneously maximizes the distance between pairs of clusters (see black lines between clusters in Fig. 2) (Khan and Ahmad, 2004). In particular, we use Partitioning Around Medoids (PAM) algorithm (Kaufman & Rousseeuw, 1990) because: (i) it is known to be one of the most robust *K*-medoids algorithm (Park & Jun 2009), and (ii) it works with continuous and discrete variables.
IV. ***Average silhouette***: we use the silhouette method (Rousseeuw, 1987) to check if the segments within a cluster were correctly classified. It relies on the silhouette width, which is a measure that relates, for every road segment, the distance to the assigned cluster against the distance to the other clusters. Higher values indicate that the algorithm correctly classified the road segment.
V. ***Optimal number of clusters***: Fridlyand (2001) claims that the average silhouette precisely measures the classification strength of the resulting clusters, with higher average silhouette values showing better clustering results. Therefore, to define the optimal number of clusters, we iteratively increase the number of clusters and select the one that maximizes the average silhouette measure (Van der Laan, Pollard, & Bryan, 2003). The optimal number of clusters might vary depending on the road network data available for the particular application.

### 3.3 Managing scalability

Given the sheer number of segments in a road network, the PAM algorithm demands significant computational resources and takes a long computational time to classify a whole city or geography (Han, Kamber, & Tung, 2001). In order to reduce the demanded resources and time requirements, we coupled the PAM algorithm with a multinomial logistic regression to predict the cluster classification of any segment in the original road network (Hosmer and Lemeshow, 2000, Starkweather and Moske, 2011).

**Fig. 4:**
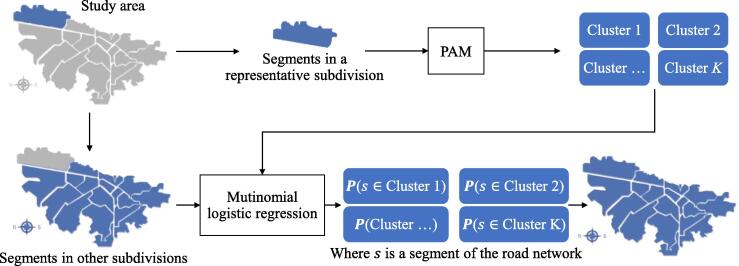
Coupling between the PAM algorithm and the multinomial logistic regression.

Fig. 4 presents a graphical example of the coupling process between PAM and the multinomial logistic regression. Instead of running the PAM algorithm over all the segments of the city-wide road network, we only use it to classify a representative subset. Depending on the case on hand, we define this subset based on relevant territorial, administrative, or political subdivisions of the road network. Then, we use the PAM algorithm to find the clusters and to classify the segments belonging to the subdivision. With the output of the PAM algorithm, we train the multinomial logistic regression to predict the likelihood of a segment to belong to the clusters found earlier by PAM. Based on the results of the multinomial logistic regression, we classify all segments of the road network efficiently.

Aside from scaling the classifier, our approach allows us to predict the impact of changes in the input variables on a given segment’s LTS classification, which may result from interventions in road conditions that may be planned or carried out. For instance, if a new street is built, the multinomial logistic regression can efficiently predict which cluster the new segment will belong to, given its expected characteristics (see Section 3.1). Or, consider a scenario where the city plans to intervene the road network, and would like to know how the planned changes would affect LTS. As these interventions may impact the characteristics of the segments, the multinomial logistic regression can easily predict changes to the level of stress of these segments.

### 3.4 Interpreting the clusters through the LTS prism

Segments classified with the same LTS category should have similar characteristics. Nevertheless, Harvey et al. (2019) demonstrated that different LTS implementations classified segments in different categories. The clustering component of our LTS-based classification methodology efficiently groups similar segments into a single cluster. After having classified all segments in a cluster, we need to interpret the cluster composition through descriptive statistics, taking into account the context of the transportation system under study. That is the main purpose of the interpretative component of our methodology. To do so, we use the LTS prism: Higher values of the width (w), number of lanes (l), average speed (v), traffic density (k), traffic flow (q), and congestion index (c) naturally imply a higher LTS. Similarly, a cluster with a larger fraction of segments with presence of heavy vehicles (h) also implies a higher LTS. On the contrary, a cluster with a large fraction of segments with cycling infrastructure (b), might imply a lower LTS (Mekuria et al., 2012). Even though our cluster analysis supports the cluster formation, it is only through a keen eye, transportation expertise, multiple perspectives, and knowledge of the local context that we can assign proper LTS labels for the road network under study. Thus, analyzing the cluster results by way of an interdisciplinary focus group or panel discussion is necessary.

We treat intersections and segments as different components of the road network, because the average stress levels inflicted on bicyclists at intersections are higher than along segments (Caviedes & Figliozzi, 2018) and because delays at intersections are major deterrents of route choice (Ryu, Chen, Christensen, & Choi, 2015). If we were to follow a similar classification approach for intersections as proposed for segments, the output clusters for intersections would very likely not coincide with those for segments. For this reason, we consider the cyclist’s perspective to classify intersections. We calculate the LTS at the intersection formed by two (or more) roadways by assigning the maximum LTS of the crossing segments.

## 4 Case study: Calculating the LTS for Bogotá (Colombia)

In this section we present the results of our data-informed LTS-based classification methodology in the road network of Bogotá. This city is home to 7.4 million inhabitants and spreads over 380 km^2^ of urban area, which makes it a metropolis that is dense, congested, with high-dense streets, and chaotic. Most of the trips in Bogotá are reported to use public transport (45%) followed by the trips made by walking and biking (25%). In the last two decades, the trips made by biking have increased significantly. The Plan Bici of Bogotá, launched in 2016, aimed at transforming cycling into the main mode of transport (Alcaldía Mayor de Bogotá & Secretaría Distrital de Movilidad, 2019). The components of the Plan Bici included institution, promotion, culture, infrastructure, safety, environment, and health. Policies such as promotional strategies, recreational events like Bogotá’s *Ciclovía* program, and implementation of protected cycling infrastructure networks are now part of the social and cultural environment of Bogotá; and have helped to increase the mode share for cycling from a mere 0.58% in 1996 to 4.5% in 2015 and perhaps as high as 9.1% in 2017 (Rosas-Satizábal & Rodriguez-Valencia, 2019).

With more than 540 km of bicycle infrastructure (Alcaldía Mayor de Bogotá & Secretaría Distrital de Movilidad, 2019), Bogotá has the most extensive bicycle infrastructure of all Latin American cities (Ríos et al., 2015). Nevertheless, despite Plan Bici’s efforts, road conditions vary substantially across the city and parts of the bicycle network are disconnected or in need of repair, affecting cyclists safety, and perception of stress. For this reason, many cyclists still prefer to use dangerous roads to move quickly between destinations and road conditions remains a major impediment to cycling uptake. Assessing cycling conditions city-wide would help improve planning practice by identifying missing connections between low-stress zones, prioritzing infrastructure, and gauging improvements. Therefore, Bogotá would highly benefit from an effective LTS indicator to enhance planning strategies and increase modal share.

### 4.1 Variables

We retrieved raw GIS data from three different sources: OpenStreetMap (OSM) (OpenStreetMap contributors, 2018), the local geospatial office IDECA (Unidad Administrativa Especial de Catastro Distrital - Colombia, 2018), and Google’s distance matrix API (Google Maps, 2018). From OSM, we retrieved the base (polyline) layer of the segments of Bogotá’s road network. From IDECA’s database we retrieved: (i) the (polygon) layer of road segments in Bogotá; (ii) the (polyline) layer of cycling infrastructure; and (iii) the (polyline) layer of bus routes of Bogotá’s Integrated Public Transport System (SITP, by its Spanish acronym). Finally, we used Google’s distance matrix API to retrieve the average and free-flow transit times for every road segment in Bogotá. To do so, we performed two queries per segment to the Google Servers. The first query retrieves the historical traversing time along the segment; and the second query retrieves the traversing time along the segment at 24:00 of Tuesday September 4, 2018, a proxy for the free-flow traversing time. At the end of this data retrieval process, we obtained data for 167,518 segments of the road network.

**Fig. 5:**
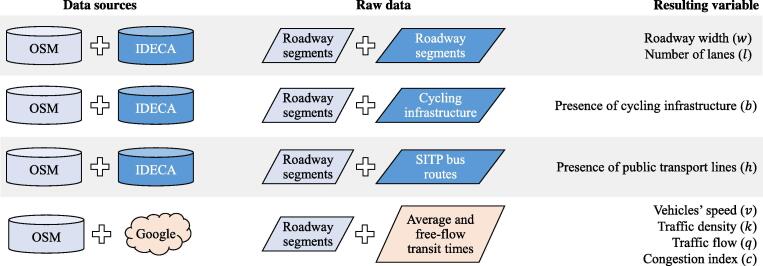
Transforming raw data into input variables.

Fig. 5 summarizes the transformation process from the raw data to the input variables that feed the LTS models. To calculate the roadway width (w) and the number of lanes (l), we used the road segment layers from OSM and IDECA. We had to combine both sources of information and check for similarities. In case they were not similar, or missing values appeared in the polyline layer from OSM, we assigned the values stored in IDECA. To identify the presence of cycling infrastructure (b), we combined OSM’s road segments layer and IDECA’s cycling infrastructure layer. We used the presence of bus routes of Bogotá’s SITP as a proxy of the presence of heavy vehicles (h). Thus, to calculate this variable, we combined OSM’s road segments layer and IDECA’s SITP bus routes layer. Therefore, we refer to this proxy variable as “Presence of public transport lines”, as it captures the presence of heavy vehicles along the road segment. Furthermore, we combined OSM’s road segments layer with Google API’s results to compute the segments’ average and free-flow transit times. With the segments’ average transit times we calculated the vehicles’ speed (v) along the segments. Having established these speeds, we computed the traffic density of every segment (Drake et al., 1967). In turn, we used this variable to calculate the traffic flow (Treiber and Kesting, 2013, chapter 7). Finally, using the average and free-flow travel times, we calculated the congestion index (Richardson & Taylor, 1978).

Many LTS implementations use a categorical variable for the type of cycling infrastructure. Nevertheless, we used a binary variable for such purpose because of data limitations. We combined the polyline layer of roadway segments from OSM with the polyline layer of cycling infrastructure from IDECA to retrieve this variable. There were 13 categories reported in IDECA that described the cycling infrastructure in a segment. Four of these categories described segments where vehicles and bicycles share the same road, representing no cycling infrastructure at all. These four categories represented <5% of the segments reported with cycling infrastructure. The remaining 95% of the segments reported with cycling infrastructure were labeled with categories that describe segregated cycling infrastructure. In total, 98.52% of these segments were labeled with categories that described segregated cycling infrastructure by a physical barrier: exclusive lane in the road segregated by bollards, exclusive lane in the sidewalk, and exclusive lane segregated with physical barriers. Because almost 93% of all the segments reported with cycling infrastructure are segregated by a physical barrier, we only considered those segments to have cycling infrastructure. Thus, we decided to use a binary variable to indicate the presence or absence of cycling infrastructure along the segments of the road network.

**Fig. 6:**
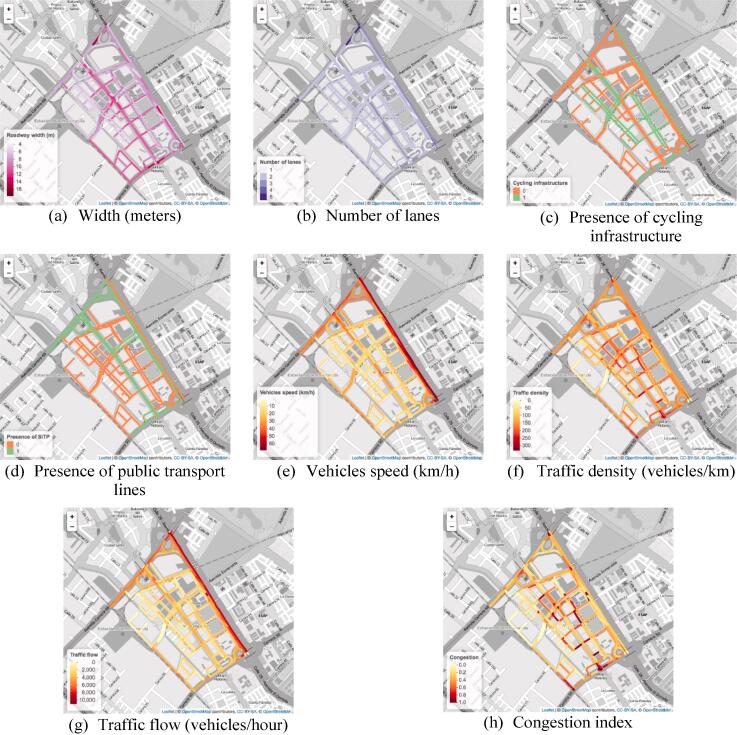
Visual display of input variables for the neighborhood of Ciudad Salitre.

Fig. 6 shows the value of the eight variables for the streets in the eastern section of the *Ciudad Salitre* neighborhood in Bogotá. We chose this neighborhood because it presents a wide range of road types. For example, it is characterized by residential streets bounded by two major streets: *Avenida Carrera 68* and *Calle 26 – Avenida El Dorado* (i.e., colored red and dark orange in Fig. 6e). It also features a major road that runs between *Carrera 50* to *Avenida Carrera 68*, through the middle of the neighborhood (*Calle 24* - *Avenida La Esperanza*). Fig. 6 shows that streets in the periphery of Ciudad Salitre tend to be wider, with more lanes, presence of public transport lines, presence of bicycle infrastructure (on Calle 26), and have more traffic flow. On the contrary, residential streets within the neighborhood tend to be narrower, without heavy vehicles or cycling infrastructure, low vehicle speed, and low traffic flow. From the visual results of our variables, we can see that streets with the most traffic density are those that connect residential streets with major streets.

### 4.2 Forming LTS clusters

**Fig. 7:**
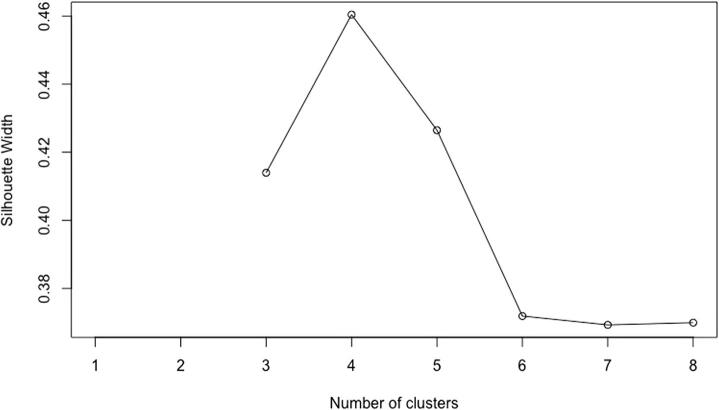
Silhouette width of the PAM algorithm after clustering segments in the locality of Usaquén.

Bogotá is divided into 20 administrative subdivisions called localities. One of the largest localities is *Usaquén*, which comprises an urban area of nearly 65.3 km^2^. We clustered the 12,887 segments in Usaquén using the PAM algorithm while maximizing the average silhouette width. Fig. 7 shows the average silhouette as a function of the number of clusters. Note that four clusters seems to be the optimal number of clusters in Usaquén.

After classifying the segments in the locality of Usaquén with the PAM algorithm, we trained a multinomial logistic regression to predict the probability of a new segment belonging to each one of the four clusters. Table 2 presents the p-values of the coefficients of the input variables over the log-odds of the classification of the road segments into each cluster with respect to Cluster 1 (base category). Most of the values were lower than 0.05 meaning that the variability of all the variables was effectively considered in the clustering, and that each feature significantly affects the likelihood of classifying a segment into a specific group. Significant coefficients imply that the average observed values of the independent variables differ between the considered cluster and Cluster 1. The p-value of the road width for Cluster 2 is not significant, meaning that Cluster 1 and Cluster 2 do not differ in the average values of road width, but do present significant differences in all the other variables.

**Table 2:** Results of the multinomial logistic regression: p-values of the variables’ coefficients.

Variable	Cluster 1	Cluster 2	Cluster 3	Cluster 4
Road width	Base category	0.298	< 0.001	< 0.001
Number of lanes		< 0.001	< 0.001	< 0.001
Vehicles' speed		< 0.001	< 0.001	< 0.001
Traffic density		< 0.001	< 0.001	< 0.001
Traffic flow		< 0.001	< 0.001	< 0.001
Congestion		< 0.001	< 0.001	< 0.001
Cycling infrastructure		0.021	0.013	0.015
Public transport lines		0.001	0.001	0.001

Using the multinomial logistic regression, we estimated the probability of a road segment belonging to each one of the four clusters for all of the 167,518 segments in Bogotá; subsequently, we classified each segment into the cluster with the largest predicted probability. Table 3 presents the average predicted probability per cluster of the segments after being classified, along with the number of classified observations. Despite the sheer number of segments, the average probability of belonging to the assigned cluster is very high. As probabilities of belonging to the other (not classified) clusters were low, we show that the multinomial logistic regression has a low chance of misclassification due to ambiguity in the selection criteria. Simultaneously, even with the high predicted probabilities, the number of classified observations is evenly distributed along all the clusters, suggesting the model exhibits no bias towards a specific group.

**Table 3:** Average probability of belonging to the classified cluster and number of classified observations.

		Average probability of belonging to cluster				
		Cluster 1	Cluster 2	Cluster 3	Cluster 4	n
**Assigned cluster**	**Cluster 1**	**1.000**	0.000	0.000	0.000	48,675
	**Cluster 2**	0.000	**0.998**	0.002	0.000	11,309
	**Cluster 3**	0.000	0.001	**0.990**	0.009	46,297
	**Cluster 4**	0.000	0.000	0.007	**0.993**	61,237

					∑	**167,518**

To assign the appropriate category of LTS to each of the four clusters, we analyzed the descriptive statistics of each cluster. Table 4 presents the summary statistics for the segments in each cluster, classified by the multinomial logistic regression. The first block of rows shows the number of segments and road kilometers. The second block of rows shows the mean and standard deviation for the continuous variables. The last block of rows presents the fraction of segments with (and without) these (categorical) attributes.

**Table 4:** Descriptive statistics for each cluster.

Metric	Clusters							
	3		1		4		2	
Number of segments	61,237		46,297		11,309		48,675	
Kilometers per cluster	2,670.19		1,906.63		473.58		1,680.30	

**Numeric variables**	**Mean**	**Std. dev.**	**Mean**	**Std. dev.**	**Mean**	**Std. dev.**	**Mean**	**Std. dev.**

Road width (m)	6.45	1.65	7.11	1.74	8.97	2.90	7.92	2.03
Number of lanes	1.96	0.21	1.95	0.33	2.31	0.83	2.07	0.50
Vehicles speed (km/h)	15.02	6.51	17.16	7.43	25.27	11.34	21.00	9.54
Traffic density (cars/km)	43.66	34.98	159.66	47.29	157.20	52.01	140.60	61.96
Traffic flow (cars/h)	611.70	568.43	2,633.70	1,127.97	3,844.00	1,922.41	2,862.00	1673.66
Congestion	0.04	0.04	0.41	0.26	0.42	0.26	0.36	0.27

**Categorical variables**	**Frac.with**	**Frac.without**	**Frac.with**	**Frac.without**	**Frac.with**	**Frac.without**	**Frac.with**	**Frac.without**

Cycling infrastructure	0.02	0.98	0.03	0.97	0.99	0.00	0.00	1.00
Public transport lines	0.00	1.00	0.00	1.00	0.89	0.12	1.00	0.00

**Fig. 8:**
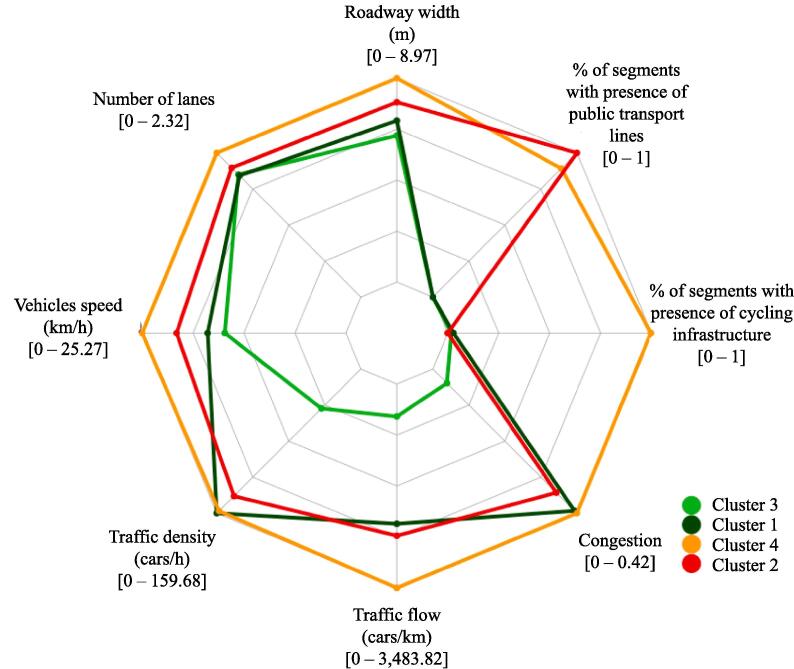
Radar plot with the average values of every variable per cluster.

Fig. 8 shows the information from Table 4 in a radar plot, with each vertex of the radar plot representing one of the eight variables. Next to the variable name, we display the range for the average value (continuous variables) and average fraction (categorical variables).

From Table 4 and Fig. 8 we note that Cluster 3 shows the lowest average values (continuous variables), reflecting narrow streets, few lanes, low speed, low traffic density, low traffic flow, and low congestion. According to the literature, low values for these variables are related with lower LTS. The segments inside this cluster also show no presence of public transport lines and lack of cycling infrastructure. The lack of heavy vehicles is commonly associated in the literature with lower levels of LTS, while the lack of cycling infrastructure could reflect that these roads are generally low-stress environments and do not require cycling infrastructure. Cluster 1 presents the largest average values for traffic density and congestion, a clear symptom of gridlock. This is consistent with the average values for street width, number of lanes, speed, and traffic flow, which are not particularly high or low. Although very few segments count with cycling infrastructure, stressful factors such as the presence of heavy vehicles are remarkably low. Putting all of these together, and relative to Bogotá’s traffic and infrastructure, a bicyclist traversing segments of Cluster 1 might experience relatively low LTS.

Clusters with variables indicating higher LTS included cluster 4 and cluster 2, which are characterized by large widths, multiple lanes, high speeds, and high traffic flow. Similarly, both clusters have a considerable fraction of segments with public transport lines. All these attributes are often associated with high levels of stress in the literature. Nevertheless, all segments inside Cluster 4 have presence of cycling infrastructure, whilst no segment in Cluster 2 does so. For this reason, we hypothesized that stressful factors in Cluster 4 could have been mitigated by the cycling infrastructure. However, stressful factors generated by traffic in Cluster 2, although not as high as those in Cluster 4, may not be relieved by any means yet.

Generally, a segment with cycling infrastructure is automatically classified as LTS 2, according to the threshold tables devised by experts (Mekuria et al., 2012). Nevertheless, in Bogotá, a vast majority of the cycling infrastructure has been built along main avenues and BRT corridors, following the theory of supply-based infrastructure planning. Therefore, these roads are far from low-stress environments, and intersections can be particularly dangerous. Nonetheless, the presence of cycling infrastructure along roads in Cluster 4 should be taken into account in the analysis that follows.

We summarized our analysis in Table 5 by using a semaphore palette, which shows from light green (not as stressful) to red (stressful) the values of the variables according to the literature. Thus, we assign the following LTS categories to the segments inside every cluster: *LTS Low* to segments inside Cluster 3; *LTS Medium* to Cluster 1; *LTS High* to Cluster 4; and *LTS Extremely high* to Cluster 2. We did not use the traditional approach of using LTS ranges from 1 to 4 because the classifications obtained for Bogotá may not be comparable to the classifications obtained with traditional LTS implementations. A segment classified as LTS Medium in Bogotá may not necessarily be associated with an LTS 2 segment following any of the available implementations of the methodology. For example, many of the segments classified as LTS Medium have low presence of cycling infrastructure, which would make them LTS 3 or 4 using traditional LTS classification; and many of the segments classified as LTS High have presence of cycling infrastructure, which would have make them LTS 2 using traditional LTS classification. Our classification ranging from LTS Low to Extremely high reflect the Bogotá-specific context, in which, due to economic factors, many cyclists need to tolerate higher stress factors and are willing to use the bicycle for their trips because, in many cases, biking is their only choice. For example, although many of the segments classified as LTS High have presence of cycling infrastructure, in reality, many cyclists even dare to use the vehicular road for their trips due to the condition of the cycling infrastructure or perceived safety, despite being exposed to more sources of stress, such as heavy vehicles and traffic speed.

**Table 5:** Assigning LTS labels to clusters. Semaphore colors shows stress from green (less stress) to red (high stress).

**Fig. 9:**
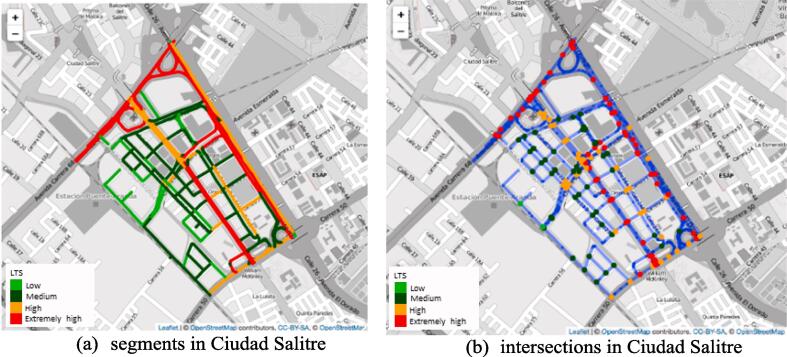
LTS intersection classification in Ciudad Salitre.

Let us focus again on the neighborhood of Ciudad Salitre (see the input variables in Fig. 6). Fig. 9A shows the LTS classification for the road segments in Ciudad Salitre. Note how the peripheral arteries Carrera Avenida 68 and Calle 26 – Avenida El Dorado, and the central avenue Calle 24 – Avenida La Esperanza, are labeled LTS *High* and LTS *Extremely high*, depending on the presence of cycling infrastructure. The residential streets within Ciudad Salitre are labeled LTS *Low* or *Medium*, with the streets that connect residential areas with main arteries all at LTS *Medium*. After classifying all segments in their proper LTS category, we calculate the LTS at intersections. Fig. 9B shows the segments in blue and the intersections as colored dots, with the color of the dot representing the LTS category at the intersection.

### 4.3 How robust is our LTS classifier?

Using different representative subsets of the road network to produce the clusters of segments could yield different LTS classifications. To check for the robustness of our data-informed LTS-based classification methodology we conducted a series of Mantel tests (Mantel, 1967) to compare the correspondence of the LTS results for the segments in a *given locality* arising from two different classifiers: (i) the unsupervised PAM results over the segments of the *given locality*; and (ii) the multinomial logistic regression predictions trained with the classified segments over a *different locality*. For this experiment, we selected three of the largest localities in Bogotá: Usaquén, *Suba*, and *Kennedy*. For the multinomial logistic regression, we trained two models using the segments from different localities. For example, if we are testing the classifiers for Usaquén, we train two multinomial logistic regression models: one with the segments of Suba, and the other with the segments of Kennedy. We applied one Mantel test per locality to compare each pair of LTS classifiers. Table 6 shows the results of the Mantel tests for each LTS category. According to these results we conclude that the LTS results from each pair of classifiers (PAM vs. multinomial logistic regression) are not statistically different.

**Table 6:** Results of the pairwise comparison of LTS classifiers by the Mantel tests (p-values).

Segments in	LTS using PAM algorithm classification	LTS using the multinomial logistic regression trained by the PAM algorithm over the segments in		
		Usaquén	Suba	Kennedy
Usaquén	LTS Low	–	0.401	0.709
	LTS Medium	–	0.257	0.912
	LTS High	–	0.912	0.559
	LTS Extremely high	–	0.257	0.709
Suba	LTS Low	0.257	–	0.401
	LTS Medium	0.401	–	0.401
	LTS High	0.146	–	0.559
	LTS Extremely high	0.709	–	0.912
Kennedy	LTS Low	0.829	0.257	–
	LTS Medium	0.559	0.146	–
	LTS High	0.829	0.829	–
	LTS Extremely high	0.401	0.912	–

Our experiment shows that, by taking as benchmark the PAM classifier, we are able to predict with the multinomial logistic regression the right LTS segment classification by using classified segments (by PAM) from other locality. This might indicate that the segment variables inside any representative locality are good predictors for the LTS classification of other locality. Since these localities are the largest in Bogotá, they comprise a wide set of diverse road segments, making them a good source to predict the right LTS for the whole road network. Hence, our data-informed LTS-based classification methodology is not only scalable but proves to be robust for the case study in Bogotá.

### 4.4 Relation of LTS and bicycle collisions

Chen et al. (2017) studied the correlation of LTS with reported bicycle crash locations for four cities in New Hampshire (US). Their results show some geospatial correlation between higher LTS segments and bicycle collisions. Similarly, we analyzed the results of our LTS classification against the fatal and non-fatal bicyclist collisions that occurred in Bogotá during 2017.

**Fig. 10:**
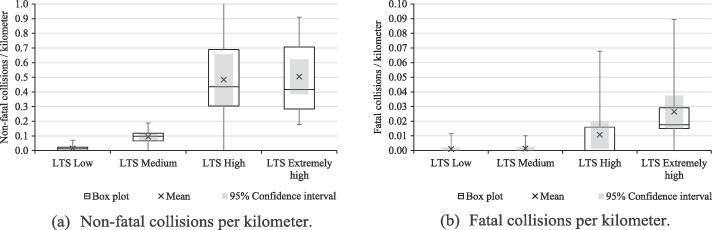
Confidence intervals of the number of injuries per kilometer of LTS.

We obtained data of collisions involving cyclists from two sources: (i) official registries of the Road Accidents Report by Bogotá’s Police Department, managed by Bogotá’s District Mobility Secretariat (SDM); and (ii) a World Resources Institute (WRI) independent road accident report based on SDM’s publicly available information. For 2017 in Bogotá, we obtained reports of 1,427 non-fatal collisions and 54 fatal collisions in the 19 urban localities that comprise the city. We geocoded each event and counted the number of fatal and non-fatal collisions in every LTS category in Bogotá. Although standardizing these collisions by exposure of cyclists is the best-practice in this kind of analyses, we did not have data on the number of cyclists that use every segment of the road network. For this reason, we standardized these collision counts by the number of road kilometers of every LTS category. Table 7 presents the mean, standard deviation, and 95% half width of these standardized counts. Fig. 10 presents the box plots and the 95% confidence intervals of the standardized collisions counts.

**Table 7:** Standardized collisions per kilometer of LTS.

	Non-fatal collisions / kilometer				Fatal collisions / kilometer			
	LTS Low	LTS Medium	LTS High	LTS Extremely High	LTS Low	LTS Medium	LTS High	LTS Extremely High
Mean	0.01505	0.09448	0.48436	0.50431	0.00094	0.00126	0.01060	0.02636
Standard deviation	0.01793	0.04718	0.35576	0.24492	0.00293	0.00311	0.01907	0.02329
Half width (95%)	0.00864	0.02274	0.17147	0.11805	0.00141	0.00150	0.00919	0.01122
Localities (data points)	19	19	19	19	19	19	19	19

From Table 7 and Fig. 10, we conclude that the number of non-fatal collisions per kilometer is higher on road segments classified as LTS High and Extremely high, than on road segments classified as LTS Low and Medium (at a 95% confidence level). Although there is no statistical difference between the number of non-fatal collisions at LTS High and Extremely high, the pattern of this number of collisions is higher than in segments classified as LTS Medium. In addition, the number of non-fatal collisions on LTS Medium segments is larger than those in roads classified as LTS Low. Regarding the number of fatal collisions per kilometer in Bogotá, this standardized count of collisions is higher at LTS Extremely high than at LTS Medium and LTS Low. In summary, these results are consistent with those reported by Chen et al. (2017)**,** as the number of fatal and non-fatal collisions per kilometer in Bogotá is statistically higher on segments labelled with higher LTS, compared to those classified as lower LTS segments.

## 5 Conclusions

In this paper, we presented a data-informed LTS-based classification methodology using readily available road network data. Our methodology involves four steps: (i) select and calculate the variables that describe the segments in the road network; (ii) calculate clusters of segments in a representative area of the road network; (iii) train a (fast) classifier and use it to assign all the segments of the city-wide road network into one of the clusters found in (ii); and (iv) assign an LTS category to each cluster based on the attributes.

Our methodology supports policymaking on at least four fronts. First, it provides planners with a context-sensitive and adaptable LTS-based classification, based on a data-informed methodology, which can form a powerful diagnostic tool for cycling network planning. Second, by using a clustering method based on physical and functional information of the road network, it avoids difficult classification decisions. Third, it allows planners to efficiently predict the impact of planned interventions on the LTS-based classification of the affected roads, according to the expected change in the input variables that describe the road network. Fourth, by grouping the intersections based on the levels obtained in the segments, yet without combining them into a single metric (which is often the case in the literature), the methodology provides an additional perspective on the stress distribution in the city. Finally, it allows for easy revision and adjustments due to the relative efficiency of the methodology.

The results of our LTS-based classification methodology produced four clusters of segments. We labeled these clusters of segments introducing LTS categories *Low*, *Medium*, *High*, and *Extremely high* that reflect the specific context of Bogotá and differentiate this LTS classification results from possible outcomes of traditional LTS implementations. A major consideration is that, despite having cycling infrastructure, segments classified as LTS High have other sources of stress such as presence of heavy vehicles or high vehicles’ speed. This is especially relevant in Bogotá’s context since some cyclist use the vehicular road for their travels regardless the presence of cycling infrastructure or its condition.

We assessed the robustness of our data-informed LTS-based classification methodology. We conducted a series of Mantel tests that showed that the four groups of segments accurately describe Bogotá’s road network in terms of the stress experienced by cyclists. Furthermore, to validate our methodology, we tallied the reported bicycle collisions in Bogotá during 2017 in each of the LTS clusters. We standardized these collision counts with the number of kilometers per LTS category at a city level. We found that there are statistically more collisions per kilometer on roads with high stress than on those with lower stress, which is consistent with the literature.

Specifically, our results support the claim that the cycling infrastructure in Bogotá has been built according to a supply-based planning method, with many major arterials and collectors featuring cycling infrastructure. However, as the collision data show, the presence of infrastructure does not mean cycling conditions along these roads are safer (though the number of cyclists should be taken into account). Moreover, despite the presence of cycling infrastructure along many arterials, the cycling network is not complete, and cyclists face high-stress conditions in many areas of the city. The cycling infrastructure may have decreased the stress of the road, but our analysis cannot assert this.

This LTS analysis will allow planners to develop new strategies and interventions to generate an interconnected low-stress network in Bogotá and a potential safer built-environment for cyclists. We expect that this methodology, although developed for Bogotá, could be applied successfully in other cities such as those included in the SALURBAL study (Quistberg et al., 2019). This LTS analysis could serve as an example to other cities willing to (1) improve cycling safety conditions; (2) improve connectivity of the cycling network by identifying missing infrastructure or lack of low-stress conditions; (3) analyze the impact of new infrastructure on cyclists; (4) prioritize infrastructure investment to areas with most potential for improvement; (5) consider alternative interventions, such as traffic calming and filtering; and (6) optimize public spending.

## CRediT authorship contribution statement

**Jorge A. Huertas:** Conceptualization, Methodology, Software, Formal analysis, Investigation, Writing - original draft, Writing - review & editing, Visualization. **Alejandro Palacio:** Methodology, Software, Formal analysis, Investigation, Writing - original draft, Writing - review & editing, Visualization. **Marcelo Botero:** Methodology, Software, Investigation, Writing - original draft, Writing - review & editing, Visualization. **Germán A. Carvajal:** Methodology, Software, Formal analysis, Investigation, Writing - original draft, Writing - review & editing, Visualization. **Thomas Laake:** Conceptualization, Methodology, Validation, Writing - review & editing. **Diana Higuera-Mendieta:** Methodology, Investigation, Writing - review & editing. **Sergio A. Cabrales:** Conceptualization, Methodology, Formal analysis, Writing - review & editing. **Luis A. Guzman:** Validation, Writing - review & editing. **Olga L. Sarmiento:** Conceptualization, Methodology, Formal analysis, Resources, Writing - review & editing, Supervision, Project administration, Funding acquisition. **Andrés L. Medaglia:** Conceptualization, Methodology, Formal analysis, Resources, Writing - review & editing, Supervision, Project administration, Funding acquisition.

## Declaration of Competing Interest

The authors declare that they have no known competing financial interests or personal relationships that could have appeared to influence the work reported in this paper.
